# Antiepileptic Drug Monotherapy: The Initial Approach in Epilepsy Management

**DOI:** 10.2174/157015909788848866

**Published:** 2009-06

**Authors:** Erik K St. Louis, William E Rosenfeld, Thomas Bramley

**Affiliations:** 1 Mayo Clinic, Rochester, Minnesota, USA; 2 St. Luke’s Hospital, St. Louis, Missouri, USA; 3 Xcenda, Salt Lake City, Utah, USA

**Keywords:** New onset epilepsy, antiepileptic drugs, monotherapy, titration.

## Abstract

Antiepileptic drug (AED) monotherapy is the preferred initial management approach in epilepsy care, since most patients may be successfully managed with the first or second monotherapy utilized. This article reviews the rationale and evidence supporting preferential use of monotherapy when possible and guidelines for initiating and successfully employing AED monotherapy. Suggested approaches to consider when patients fail monotherapy include substituting a new AED monotherapy, initiating chronic maintenance AED polytherapy, or pursuit of non-pharmacologic treatments such as epilepsy surgery or vagus nerve stimulation. Reducing AED polytherapy to monotherapy frequently reduces the burden of adverse effects and may also improve seizure control. AED monotherapy remains the optimal approach for managing most patients with epilepsy.

## INTRODUCTION

Epileptic seizures have been observed since antiquity [57]. Treatment preferences generally favored polytherapy prior to the evolution of modern antiepileptic drugs (AEDs). In the early 1900s, phenobarbital and the ketogenic diet were used to manage epilepsy. Throughout the earlier 20^th^ century, the standard AEDs (phenytoin, phenobarbital, primidone, valproic acid, carbamazepine, and ethosuximide) were often combined in polytherapy use, due to the pervasive belief that polytherapy was more efficacious than monotherapy. However, during the 1970s, several studies suggested that monotherapy was equally efficacious, less toxic, and more tolerable than polytherapy [51,52]. Since then, most epilepsy experts have advocated monotherapy as the preferred approach in epilepsy, although polytherapy is sometimes still necessary. This review examines the evidence favoring initial monotherapy and suggests methods to maintain monotherapy or reduce polytherapy to monotherapy when possible.

## THE RATIONALE FAVORING MONOTHERAPY

Since the early 1990s, the second-generation AEDs, felbamate, gabapentin, lamotrigine, topiramate, tiagabine, oxcarbazepine, levetiracetam, zonisamide, and pregabalin, have become available. Recently, the seemingly ever increasing armamentarium of AEDs has seen two additional newer (“third-generation”) AEDs released, lacosamide and rufinamide. Advantages of most newer AEDs include a more desirable safety profile and fewer adverse effects and drug interactions than their predecessors. Recent pivotal clinical trials have provided evidence to support monotherapy use of second-generation AEDs [42]. Current treatment guidelines recommend monotherapy in most cases because data indicate similar efficacy and better patient tolerability compared to polytherapy [43-45,48,49,51,52]. Polytherapy may only minimally increase seizure control and can substantially increase AED toxicity, [9,20,43-45,48,49,51,52] drug interactions, [2,21,30,34,38,39,40,41] seizure aggravation [43,44], comorbid depression,[39] risk of sudden unexplained death in epilepsy patients (SUDEP), [28,36,37] noncompliance, [8] and cost [4]. Polytherapy and seizure burden were the two main causes of quality of life impairment in one recent survey of epilepsy patients [58].

## WHO BENEFITS MOST FROM MONOTHERAPY?

While monotherapy is preferable for most patients with epilepsy, monotherapy is particularly desirable for certain special patient populations, including women, elderly, and patients with co-morbid conditions (who are at increased risk for AED toxicity and drug interactions) [11,21,38]. Compared to polytherapy, monotherapy reduces the potential for adverse drug interactions. Hepatic and renal dysfunction significantly impacts the metabolism and elimination of many AEDs, which may reduce tolerability and safety of continued use. Pregnant women taking two or more AEDs are at substantially increased risk of fetal malformations (3% versus 15%) than mothers receiving monotherapy [11]. See Table **1** for a list of monotherapy recommendations.

## EVIDENCE SUPPORTING PREFERENTIAL MONOTHERAPY IN EPILEPSY

The majority of patients with epilepsy respond to treatment with monotherapy; 47% of patients become seizure-free with the first AED tried, and another 13% achieve freedom from seizures with the second monotherapy trial [24].

While available evidence is central in determining whether an AED is effective for monotherapy usage, FDA approval and indication generally guide how an AED will be prescribed. First-generation AEDs were “grandfathered” by the FDA, receiving approval for monotherapy for a particular seizure type without requirement to satisfy current rigorous approval requirements [42]. The majority of second-generation AEDs are approved only as adjunctive therapies. Current FDA standards require superiority trial designs since placebo-controlled studies are considered unethical in epilepsy, and few such studies have been conducted given the practical difficulties and expense involved in such trials [42]. United States practitioners are thus at the mercy of superiority trial design data produced by trials that are conducted for the purpose of gaining FDA approval, a somewhat artificial circumstance leaving practitioners in doubt as to how to utilize the AED for monotherapy in clinical practice, unlike in Europe where approval standards permit the more practical standard of equivalence trial designs. As a result, many U.S. clinicians often continue to prescribe first-generation AEDs for new onset epilepsy because of experience and familiarity, limited comparative efficacy data with newer AEDs, and concern over the higher cost of newer drugs. To determine the best AED choices for monotherapy, further randomized, double-blind, long-term, comparative clinical trials with the newer AEDs are needed. Since few comparator studies are funded by industry, government agencies should become involved in conducting additional comparative clinical trial studies, and independent groups (ie, International League Against Epilepsy) should be persuaded to collect data from historically treated and control patients.

Currently, four second-generation AEDs are FDA approved for use as monotherapy, with some limitations; these are oxcarbazepine, lamotrigine, topiramate, and felbamate [17]. In four randomized, controlled, blinded trials, oxcarbazepine demonstrated efficacy as monotherapy in patients with partial seizures [36,60]. Lamotrigine is currently approved as monotherapy when converting from an enzyme-inducing AED or valproate but not for de novo or initial monotherapy [17]. However, lamotrigine should be used with caution in persons under the age of 16 due to a higher incidence of a potentially life-threatening rash in pediatric patients, [27] and patients receiving concurrent valproic acid or who receive inappropriately fast initial titration of lamotrigine are also at heightened risk of serious rash [34]. Topiramate is indicated as initial monotherapy in adults and children aged 10 years and older with partial onset or primary generalized seizures; efficacy was established in both a large, double-blind, dose-controlled study and a second large trial comparing two doses of topiramate with standard comparators carbamazepine and valproate [3,17,59]. Felbamate also has evidence for monotherapy use in partial-onset seizures; however, severe idiosyncratic toxicities limit its use [15,16]. Additionally, gabapentin possesses adequate evidence for confident use as monotherapy in treatment of partial-onset seizures, although it lacks formal FDA approval for this indication [10,17].

Among the second-generation AEDs approved for monotherapy use, few comparator trials have been conducted. Gabapentin and lamotrigine have been shown to be comparably effective and tolerable in two large prospective trials, and were more tolerable than carbamazepine in elderly with newly diagnosed epilepsy [6]. A large, naturalistic, unblinded controlled trial recently demonstrated superior efficacy (for time to treatment failure) of lamotrigine as compared to carbamazepine, oxcarbazepine, and topiramate [31].

The other second-generation and third-generation AEDs have not been FDA approved for monotherapy use since most lack an adequate level of evidence for this indication. However, the efficacy and tolerability of levetiracetam monotherapy for treatment of partial-onset seizures has been confirmed in a recent large, prospective, comparator trial against carbamazepine [7]. Small controlled clinical trials are also available to support tiagabine monotherapy use [47].

## GUIDELINES FOR AED MONOTHERAPY

Given the complexity and expansive body of evidence concerning AED therapy in the medical literature and limitations in practical application of this literature to actual patients, practice guidelines and expert surveys are valuable tools to assist clinicians in applying evidence based practice for patients with epilepsy. Practice guidelines are available to assist practitioners in the management of new-onset and refractory epilepsy and epilepsy in women. The American Academy of Neurology/American Epilepsy Society (AAN/AES) Practice Guidelines for the treatment of new-onset epilepsy identified gabapentin, lamotrigine, oxcarbazepine, and topiramate as possessing Class I evidence (prospective study, blinding, statistical population-based sample, and patients studied concurrently and early in the course of therapy) for use as monotherapy in the treatment of new-onset partial or mixed seizures [18]. The recently updated guidelines for the treatment of epilepsy in women state that in women with epilepsy (WWE), monotherapy is recommended during the reproductive years to reduce the risk of teratogenicity seen with polytherapy [1].

A recent survey of epilepsy experts found that lamotrigine, levetiracetam, and valproic acid are preferred AED choices for monotherapy in the treatment of generalized tonic-clonic, absence, and myoclonic seizures [23]. Previous survey results were compared to the current survey, and, overall, valproic acid is still the drug of choice for each of these seizure types, except for absence seizures, where ethosuximide remains the preferred AED. Many practitioners chose lamotrigine and topiramate as first-line treatment for generalized tonic-clonic seizures.

## HOW TO INITIATE MONOTHERAPY

Practical tenets for achieving successful monotherapy in new-onset epilepsy management include the following: 1) select an efficacious AED for the specific seizure type; 2) choose an AED with a tolerable adverse effect and toxicity profile; and 3) titrate the AED slowly to the desired dose, taking into account the patient’s response to treatment. If the first AED monotherapy is ineffective, adding a second AED, then tapering and discontinuing the ineffective AED, is the preferred approach [56]. When switching AEDs, selecting an agent with a different MOA may increase the likelihood of a successful treatment response. If the second sequential AED monotherapy is ineffective, an adjunctive AED with a different and potentially complementary MOA should be considered for use in adjunctive polytherapy. Since approximately 35% of patients with epilepsy will not respond to monotherapy, most refractory patients become candidates for polytherapy [24,26]. Polytherapy with lower or moderate dosages of two AEDs may also sometimes be preferred for management of refractory patients who have dose-limiting neurotoxic adverse effects with high-dose monotherapy [24].

Before initiating treatment, patients with epilepsy should undergo a thorough medical evaluation to determine seizure type and consider baseline patient characteristics that may influence the decision of whether treatment is necessary and, if so, which AED may be the most logical choice. Evaluation to determine the patient’s epilepsy syndrome begins with a thorough clinical history, including a detailed description of the seizure semiology, an awake and asleep electroencephalogram (EEG), and a brain magnetic resonance image (MRI). A standard brain MRI is adequate in newly diagnosed epilepsy where the priority is to exclude underlying serious symptomatic pathologies, such as arteriovenous malformations or neoplasms. However, when feasible, available, and of reasonable cost, it is advantageous to consider obtaining brain MRI with a volumetric seizure-protocol study in patients with suspected partial-onset seizures, given better identification of subtle mesial temporal lobe pathologies such as hippocampal sclerosis or malformations of cortical development that may impact on prognosis for drug responsiveness, as well as future decisions regarding surgical triage if the patient becomes refractory to AEDs.

Educating the patient about epilepsy, AED compliance, and seizure first-aid is important for ensuring successful therapy. AED selection is determined by seizure type, patient medical history, and concurrent medications. For partial epilepsy, any approved AED could be considered for use (except ethosuximide, which is ineffective for partial-onset seizure treatment). In idiopathic or symptomatic generalized epilepsies, as well as for ambiguous or unknown epilepsy syndromes, a broad-spectrum AED should be preferentially utilized given the potential to treat (or avoid aggravation of) other potentially associated generalized seizure-types in generalized epilepsies (i.e., absence and myoclonic seizures in idiopathic generalized epilepsies, or tonic seizures in symptomatic generalized epilepsy).

Once an AED has been selected, starting with a low dose and gradually titrating to a moderate and presumably effective dose is a reasonable strategy, since titration to doses higher than a mean effective dosage are often poorly tolerated and only provide additional efficacy in perhaps 10% to 15% of patients [24]. A more rapid titration may be necessary in selected patients who have had multiple recent seizures. Maximizing the dose is only recommended in patients who do not respond to moderate doses. At each patient visit, it is necessary to conduct a detailed assessment of AED therapy, including adverse effects and compliance. Utilizing a quantitative survey of patient’s perceived adverse effects such as the adverse events profile (AEP) has been shown to improve physicians’ ability to detect adverse effects of treatment and appropriately alter antiepileptic drug therapies to improve quality of life [19]. The impact of adverse effects on daily living should be discussed, and any barriers to compliance should be addressed.

## WHY ARE SOME PATIENTS REFRACTORY TO MONOTHERAPY?

The efficacy of currently available AEDs is limited to reduction of seizure frequency, and no AED has yet been proven to impact the pathophysiology of epilepsy itself [55]. Currently, there is no convincing evidence that any of the available AEDs are anti-epileptogenic, nor has any AED been shown to favorably impact the long-term outcome of epilepsy [24]. Yet, prescribing an AED after a second or third seizure event is the accepted practice standard, based on logic derived in part from epidemiologic studies of the natural history of new onset unprovoked seizures that confirm a greatly increased risk for further seizure recurrence following the occurrence of a second unprovoked seizure [22]. The desired short-term outcomes of epilepsy management are seizure freedom, seizure control when complete seizure freedom is not possible, and maximizing patient quality of life, given that the complications of untreated seizure activity are increased risk of injury and mortality, cognitive and behavioral abnormalities, and social disadvantage. Currently, there is no AED for the treatment of epilepsy that is completely effective, without adverse effects, and efficacious for all patients. Of the older AEDs, carbamazepine was shown to be the most effective and tolerable AED in two pivotal clinical trials; thus, it became the standard to which developing AEDs have been compared, [7,31-33] although carbamazepine itself has never been compared to placebo or demonstrated efficacy in an active control-designed study. Most studies have shown that newer AEDs have equivalent efficacy to that of carbamazepine, but several newer AEDs have superior tolerability including lamotrigine and gabapentin [6,46]. However, recently published comparator trials of newer AEDs against a sustained release form of carbamazepine have shown relatively equivalent tolerability, suggesting that immediate release forms of carbamazepine may be less tolerable and sustained release forms of carbamazepine are equally tolerable to newer AEDs [7,31].

## WHY DOES MONOTHERAPY FAIL?

AED monotherapy may fail for a variety of reasons, including errant diagnosis (eg, mistaking syncopal spells as seizures), inaccurate diagnosis of seizure type leading to ineffective AED choice (eg, mistaking partial complex seizures for absence with prescription of carbamazepine rather than ethosuximide or valproate), intolerable adverse effects (eg, depression, sedation, cognition problems), idiosyncratic reactions (eg, rash, aplastic anemia, hepatoxicity), noncompliance, over treatment, [50] and pharmacogenetic factors [53]. Monotherapy is most likely to be effective if the clinician develops a personalized treatment plan that is appropriately customized for the individual patient, provides the patient with suitable education concerning the drug chosen, and offers the opportunity for close telephone follow-up and surveillance with evolution of any adverse effects to enable prompt feedback and modification of the titration scheme or target dose.

Monotherapy may be ineffective when the AED choice is suboptimal for a particular patient type. Certain AEDs are arguably best avoided in certain patient types, such as phenytoin or carbamazepine in the elderly [21,29] (given their vulnerability for adverse effects, osteoporosis, and drug interactions) or valproate in women of child-bearing potential (given its heightened teratogenic risk) [11]. AEDs that require substantial hepatic metabolism are best avoided in patients with liver disease. Carbamazepine, phenytoin, phenobarbital, primidone, oxcarbazepine, and high-dose topiramate are enzyme inducers; these agents increase the hepatic metabolism of concurrently administered drugs, endogenous and exogenous sex hormones, and vitamin D. Enzyme-inducing AEDs can lead to: therapeutic failure of other inducible AEDs and other drugs such as anticoagulants, hormonal contraceptives, lipid-lowering agents, and antihypertensives; reproductive dysfunction; and osteopenia or frank osteoporosis [21,40,41]. Primidone and phenobarbital are not commonly recommended as first-line therapies due to poor tolerability and efficacy as well as abuse potential. Felbamate is associated with rare but potentially fatal idiosyncratic hematologic and hepatic toxicities, limiting its use solely to brittlely refractory patients [16].

Overtreatment may be another cause of failure [14]. Approximately 50% of patients with new onset epilepsy achieve remission with moderate doses of the first AED prescribed [26]. When a moderate dose fails, maximizing the dose results in up to 10-25% of patients obtaining seizure control, [25] but at the risk of adverse events. Rarely, some epilepsy patients may experience a paradoxical increase in seizure frequency or severity at higher AED doses [54]. If no improvement or a worsening in seizure activity is seen, the AED dose should be reduced or the drug discontinued.

## WHAT TO DO WHEN MONOTHERAPY FAILS

Patients who do not achieve seizure freedom on an appropriately selected and optimally administered initial AED monotherapy are unlikely to become seizure free on future AED trials [25]. If the patient is experiencing breakthrough seizures while receiving moderate doses of an AED, increasing the dosage of that AED until the patient has either become clinically toxic or has shown a clear plateau in response to the medication is reasonable. If the patient has continued breakthrough seizures at this point, transition to a second monotherapy is indicated. Approximately two-thirds of epilepsy patients will have adequate seizure control with either the first or second trial of AED monotherapy [25]. The remaining third are considered to have refractory epilepsy. If a second monotherapy also fails, consideration of initiating AED polytherapy by adding a drug with a different and complementary principle mechanism of action should be considered. While monotherapy is preferable to polytherapy, some patients will require more than one AED to attain successful seizure control [12,13]. In refractory patients receiving polytherapy, the treatment goal necessarily shifts somewhat from the goal of producing seizure freedom, which is increasingly improbable, to effecting palliation and control of seizures while minimizing adverse effects. Since refractory patients rarely attain seizure-freedom with polytherapy, many require evaluation for alternative non-pharmacologic treatments such as resection surgery or vagus nerve stimulation (VNS).

Patients who subsequently become refractory to the first or second empiric AED monotherapy should be strongly considered for ictal seizure recording with video-EEG monitoring to ensure that the epilepsy diagnosis is accurate, and if so, to establish the correct epilepsy syndrome diagnosis. Video-EEG monitoring allows an accurate diagnosis of the seizure-type, which may enable the clinician to appropriately tailor AED therapy and explore the patient’s potential candidacy for resection surgery or VNS therapy. Video-EEG monitoring also ensures exclusion of diagnoses that mimic epilepsy, such as psychogenic non-epileptic spells (ie, pseudoseizures), a particularly common finding representing between 30-50% of admissions to most epilepsy monitoring units. In such cases, AEDs may in most cases be tapered and discontinued, with referral to appropriate psychological counseling resources, and when necessary, psychiatric care. More rarely, physiologic non-epileptic spells such as syncope or sleep disorders are instead identified in some patients with suspected epilepsy, and appropriate referral to other specialists can be offered.

## CONVERTING FROM POLYTHERAPY TO MONOTHERAPY

Patients may begin receiving polytherapy while transitioning from one trial of monotherapy to another (transitional monotherapy), or because of two failed attempts with monotherapy (chronic polytherapy). In the first situation, eventual monotherapy is likely to result once the original AED is tapered off. However, in the latter case, patients may continue on multiple AEDs indefinitely. While receiving multiple AEDs, some patients may go into seizure remission, while other patients will continue to require sequential trials of additional AEDs (AED sequencing). Patients who are appropriate candidates for tapering one or more AEDs are those who have been seizure free for 2 years or longer. For these patients, a slow taper is recommended, with dose reductions occurring weekly or every other week. Additionally, patients receiving unsuccessful polytherapy (ie, polytherapy that has failed to produce seizure freedom or that is resulting in intolerable adverse effects) should be considered for a further trial of monotherapy or additional AED sequencing.

## CONCLUSIONS

Epilepsy treatment often requires lifelong medication management. Monotherapy is preferred when managing patients with epilepsy, given similar efficacy and superior tolerability compared to polytherapy for most patients, especially those with newly diagnosed epilepsy who are not refractory to other treatments. While preferred for initial use in epilepsy treatment, monotherapy may also fail for a variety of reasons. Monotherapy may fail in patients who do not receive thorough evaluation and counseling by their physician. Patients need to have a clear understanding of treatment expectations candidates for tapering of one or more AEDs. Adverse effects, noncompliance, and evolving refractory epilepsy are the principle reasons for treatment failure. To increase the likelihood of successful monotherapy, clinicians should consider individual patient characteristics, including seizure type, potential drug interactions, likelihood of compliance, and cost, while realizing that therapy may require modification. Vigilance for need of further medication titration or tapering, the patient’s current seizure frequency and severity, and occurrence of adverse effects is necessary for successful monotherapy in epilepsy care.

## Figures and Tables

**Table 1.** Monotherapy AED Options for Different Patient Populations: A Compilation of Practice Guidelines and Clinician Recommendations for the Treatment of Generalized Tonic-Clonic, Absence, Partial, and Myoclonic Seizures*‡†

Patient Characteristic	First Line	Supporting Reference
Elderly	Lamotrigine or Levetiracetam	[26, 27]
Female of reproductive age	Lamotrigine	[25-27]
Pregnant	Lamotrigine	[26, 27]
Liver failure	Levetiracetam or Lamotrigine or Gabapentin^a^	[26, 27]
Renal failure	Lamotrigine or Valproic acid^b^	[26, 27]
Depression	Lamotrigine or Valproic acid^b^ or Oxcarbazepine^c^	[26, 27]

a Absence seizures only.

b Generalized tonic-clonic seizures only.

c Simple partial and secondarily generalized tonic-clonic seizures only.

* Pregabalin not available at time of these studies.

‡ Topiramate not recommended in any of these patient groups by Karceski and colleagues [23]; however, French *et al.* [17] recommends topiramate as monotherapy.

† French *et al.* [17] evaluated second-generation AEDs only.
